# Comparison of admission serum albumin and hemoglobin as predictors of outcome in children with moderate to severe traumatic brain injury: A retrospective study: Erratum

**DOI:** 10.1097/MD.0000000000018562

**Published:** 2019-12-20

In the article, “Comparison of admission serum albumin and hemoglobin as predictors of outcome in children with moderate to severe traumatic brain injury: A retrospective study”,^[1]^ which appears in Volume 98, Issue 44 of *Medicine*, affiliation a should appear as “Department of Infectious Disease, the First Affiliated Hospital of Chongqing Medical University, Chongqing, China.”
